# High Incidence of Oxacillin-Susceptible *mecA*-Positive *Staphylococcus aureus* (OS-MRSA) Associated with Bovine Mastitis in China

**DOI:** 10.1371/journal.pone.0088134

**Published:** 2014-02-11

**Authors:** WanXia Pu, Yang Su, JianXi Li, ChunHui Li, ZhiQiang Yang, HaiPing Deng, ChunXia Ni

**Affiliations:** Key Laboratory of New Animal Drug Project, Gansu Province, Key Laboratory of Veterinary Pharmaceutical Development, Ministry of Agriculture, Lanzhou Institute of Husbandry and Pharmaceutical Sciences of CAAS, Lanzhou, China; Iowa State University, United States of America

## Abstract

*Staphylococcus aureus* is a main cause of bovine mastitis and a major pathogen affecting human health. The emergence and spread of methicillin-resistant *Staphylococcus aureus* (MRSA) has become a significant concern for both animal health and public health. This study investigated the incidence of MRSA in milk samples collected from dairy cows with clinical mastitis and characterized the MRSA isolates using antimicrobial susceptibility tests and genetic typing methods. In total, 103 *S. aureus* isolates were obtained from dairy farms in 4 different provinces in China, including Gansu, Shanghai, Sichuan, and Guizhou. Antimicrobial susceptibility testing of these isolates revealed that the resistance rates to penicillin and sulfamethoxazole were high, while the resistance rates to ciprofloxacin and vancomycin were low. Among the 103 isolates, 49 (47.6%) were found to be *mecA*-positive, indicating the high incidence of MRSA. However, 37 of the 49 *mecA*-positive isolates were susceptible to oxacillin as determined by antimicrobial susceptibility assays and were thus classified as oxacillin-susceptible *mecA*-positive *S. aureus* (OS-MRSA). These isolates could be misclassified as methicillin susceptible *Staphylococcus aureus* (MSSA) if genetic detection of *mecA* was not performed. Molecular characterization of selected *mecA*-positive isolates showed that they were all negative with Panton-Valentine leukocidin (PVL), but belonged to different *spa* types and SCC*mec* types. These results indicate that OS-MRSA is common in bovine mastitis in China and underscore the need for genetic methods (in addition to phenotypic tests) to accurately identify MRSA.

## Introduction

*Staphylococcus aureus* causes infections in both people and animals. Methicillin-resistant *S. aureus* (MRSA) is a prominent pathogen in nosocomial and community acquired infections, and is a major threat to human health worldwide due to its antimicrobial resistance, infectivity and possession of virulence factors [1]–[5]. Methicillin resistance in *S. aureus* is mainly mediated by the expression of the *mecA* gene, which is located on a mobile genetic element, staphylococcal cassette chromosome *mec* (SCC*mec*), and encodes an altered penicillin-binding protein (*PBP2a*) with an extremely low affinity to β-lactam antibiotics, making it possible for *S. aureus* to survive the treatment of β-lactam antibiotics [6]. *S. aureus* that either have the *mecA* gene or show a minimum inhibitory concentration (MIC) of oxacillin higher than 4 µg/ml are defined as MRSA. However, *S. aureus* that are positive for *mecA* and *PBP2a*, but phenotypically susceptible to oxacillin have been reported [7]–[9]. It is generally accepted that such *S. aureus* isolates should be defined as oxacillin-susceptible *mecA-*positive *S. aureus* (OS-MRSA). Identification of OS-MRSA has clinical implications as precautions have been proposed for the treatment of OS-MRSA infections. Although OS-MRSA is phenotypically susceptible to oxacillin, it may be prone to the development of highly resistant MRSA under antibiotic selection due to the possession of *mecA* [10] [11].

China has recently experienced significant growth in dairy industry and the total milk production in China reached to the third place worldwide in 2012 [12]. In China, bovine mastitis is a serious problem for dairy industries and the average incidence rate is about 33%, incurring considerable economic losses [13]. For example, it is estimated that 5–10% cows are culled annually in the Shanghai region due to mastitis [14]. Although multiple pathogens are associated with bovine mastitis, *S. aureus* is a common and important cause of the disease [15]. Antimicrobial treatment is often used to decrease the incidence or shorten the duration of bovine mastitis; however, treatment failure occurs due to development of antibiotic resistance [16].

Despite the fact that *S. aureus* is commonly associated with bovine mastitis, MRSA isolates have been infrequently reported with the disease. There have been a few reports of MRSA colonization and/or infections in dairy cattle since the very first report of MRSA in mastitis in 1972 [17]–[21]. Recently, a highly divergent *mecA* gene (now named *mecC*) in a typeXI SCC*mec* was found in bovine mastitis S. *aureus* [22] [23]. Mastitic MRSA strains from different continents may share similar or different molecular characteristics. For example, reports from some European countries indicated that ST398 MRSA with SCC*mec* type IV or V played an important part in clinical or subclinical bovine mastitis, although it was not the only clonal lineage associated with mastitis [24]. Several genotypes including ST1/t286 MRSA with SCC*mec* type IVa, ST72/t324 MRSA with SCC*mec* type IV or IVa, and ST72/untypeable *spa*-type with SCC*mec* type IV were reported in Korea [25]. The majority of reported MRSA isolates in Turkey belonged to ST239/*spa*-type t30 with SCC*mec* type III, while others belonged to ST8/*spa*-type t190/SCC*mec* type IV, or ST329/*spa*-type t30/SCC*mec* type III [26]. These data indicated that various MRSA clones or genotypes were associated with bovine mastitis in different countries.

In China, there have been a few reports on MRSA of animal origin. Cui et al. reported presence of MRSA from swine and swine farm workers in four Chinese provinces, all of which belonged to ST9 and *spa* type t899, contained a type III SCC*mec* element, and lacked the *Panton-Valentine Leukocidin (PVL) gene* [27]. There was a report on MRSA from pet animals and veterinary staff in China, in which 22 MRSA isolates were identified by using the API Staph Ident System, MIC tests and *mecA*-specific PCR assay [28]. Another study reported that MRSA of ST97 with SCC*mec* typeIV, ST965 with SCC*mec* typeIV, ST6 with SCC*mec* typeIV and ST9 with untypeable SCC*mec* were found in milk samples collected from bovine mastitis cases [29].

Despite the numerous reports of MRSA of human and animal origins in different countries, there have been few published studies on OS-MRSA associated with bovine mastitis. Due to the rising significance of OS-MRSA in clinical treatment, enhanced efforts are needed to identify this type of MRSA by combining phenotypic tests with genetic methods. The objective of this study was to determine the incidence of OS-MRSA in bovine mastitis and to characterize the MRSA isolates using various methods.

## Materials and Methods

### 1. Ethics

Milk samples were obtained on farms from dairy cows with naturally occurring clinical mastitis with consent from farm owners under the ethical approval by Lanzhou Institute of Husbandry and Pharmaceutical Sciences of CAAS. The collections were done by professional veterinarians and permitted by the owners of the dairy farms under investigation. Conventional milking methods were used and no invasive or pain-causing procedures were involved. This study did not involve endangered or protected species and did not use animals for experiments.

### 2. Sample collection and bacterial isolation

Milk samples were taken from cows with clinical mastitis which was manifested with decreased milk production, color change of the milk, and inflammation of the udder. These samples were collected from farms in 4 different and geographically diverse regions in China including Gansu, Shanghai, Sichuan, and Guizhou in 2008. These farms represent typical dairy production practices in China. For milk collection, udders of the clinical mastitis cow were washed with clean water and dried. Cotton swabs soaked with 70% ethanol were used to disinfect the surfaces of teats. The first few streams of milk were discarded. Then a milk sample was collected into a 10-ml sterile plastic tube. The collected samples were kept in a cooler with ice and transported to the laboratory within 8 hours. Then the samples were stored at −20°C for bacterial isolation.

Isolation of *S. aureus* was done in the Key Laboratory of Veterinary Pharmaceutical Development, Ministry of Agriculture, Lanzhou, China. Briefly, the milk samples were inoculated on 5% sheep blood agar plates and inoculated at 37°C for 24 h. S. *aureus* identification was based on Gram staining, morphology, and traditional biochemical tests, including catalase, coagulase and mannitol fermentation tests [30] [31].

In total, 103 *S. aureus* isolates were obtained, including 17 from Gansu, 52 from Shanghai, 16 from Sichuan, and 18 from Guizhou. The 17 isolates from Gansu were isolated from 150 clinical mastitic milk samples, which were collected from three different commercial dairy farms (50 samples per farm). The 52 *S. aureus* strains from Shanghai were isolated from 200 milk samples of two different farms (100 samples per farm); the 16 isolates from Sichuan were derived from 50 milk samples; and the 18 isolates from Guizhou were obtained from 50 milk samples. All *S. aureus* isolates were stored at −80°C. *S. aureus* ATCC43300 and ATCC25923 were used as quality control organisms.

### 3. Phenotypic identification of MRSA

*S. aureus* isolates were tested for methicillin resistance using the cefoxitin and oxacillin disk diffusion methods outlined by the Clinical and Laboratory Standards Institute [32]. Cefoxitin disk (30 µg, Oxoid) and oxacillin disk (1 µg, Oxoid) were used in this study. The zones of inhibition were measured after 18–20 hours incubation at 35°C. Isolates with zone sizes less than 21 mm for cefoxitin and 10 mm for oxacillin were considered methicillin resistant according to the criteria of CLSI [32].

### 4. Antimicrobial susceptibility tests

The *mecA*-positive MRSA isolates were tested for susceptibility to various commonly used antimicrobial agents by using two different methods. The disk diffusion test was performed with penicillin (10 IU/disk), gentamicin (10 µg/disk), tetracycline (30 µg/disk), erythromycin (15 µg/disk), ciprofloxacin (5 µg/disk), sulfamethoxazole (300 µg/disk), cefazolin (30 µg/disk) and clindamycin (2 µg/disk). The agar dilution method was used to measure the MICs of vancomycin and oxacillin. Both disk diffusion and agar dilution were performed according to the recommendations of CLSI [32]. The breakpoints of CSLI for the tested antibiotics (for both disk diffusion and agar dilution) were used to determine the susceptibility profiles. All antimicrobial susceptibility testing assays were repeated at least 3 times.

### 5. Genotypic identification of MRSA

A single colony of *S. aureus* was inoculated into LB culture medium, and the culture was shaken overnight at 37°C. Then the culture was used for preparation of genomic DNA using the TIANamp Bacteria DNA Kit [TIANGEN BIOTECH (Beijing) CO., LTD] according to the manufacturer's instructions. The genomic DNA was used as template for PCR for typing of SCC*mec* and *spa* and for detection of *mecA* and the *PVL* toxin gene (*lukF-lukS*).

The primers used to amplify the *mecA* gene (310 bp) of MRSA were P1: 5′-TGGCATTCGTGTCACAATCG-3′ and P2: 5′-CTGGAACTTGTTGAGCAGAG-3′ as previously described [33]. Each PCR mixture was composed of 2 µl DNA template, 0.5 µl of each primer (10 µM), 12.5 µl ExTaq buffer mix [TaKaRa Biotechnology (Dalian) Co., Ltd], and 9.5 µl sterile distilled H_2_O. PCR program began with an initial denaturation step at 94°C for 4 min followed by 34 cycles of 92°C for 1 min, 53°C for 50 seconds, and 72°C for 1 min, and ended with a final extension step at 72°C for 10 min. The *mecA*-positive strain ATCC43300 and the *mecA*-negative ATCC25923 were included as positive and negative controls, respectively. The amplified PCR products were electrophoresed in 2% agarose gel at 120 V for 1 hour, stained with ethidium bromide (0.5 µg/ml), and photographed under UV light.

### *6. Spa*-typing

Using the Ridom StaphType standard protocol(www.ridom.com), the MRSA strains from Gansu and Shanghai were PCR amplified for analyzing the polymorphic X-region of *Staphylococcus* protein A (*spa*) gene. The amplicons were purified using a TIANgel Midi Puification Kit [TIANGEN BIOTECH (Beijing) CO., LTD] and sequenced using the same PCR primers at Sangon Biotech (Shanghai) Co., Ltd. The *spa* types were assigned by using an online *spa* database [34].

### 7. SCC*mec* typing

SCC*mec* types were determined using the primers as described by Zhang et al [35]. A combination of different PCR reactions was performed to type the SCC*mec* elements. For specific SCC*mec* types, the SCC*mec* M-PCR typing assay contained 2 pairs of primers including one pair specific for the *mecA* gene and another pair specific for SCC*mec* types and subtypes I, II, III, IVa, IVb, IVc, IVd and V. Then a single PCR reaction was performed to identify the related *mec* and *ccr* gene complexes using specific primers. In addition, the PCR amplicon of SCC*mec* of isolate B5 was sequenced and compared with the standard SCC*mec* sequences in the NCBI database.

### 8. Detection of the Panton-Valentine Leukocidin gene *lukF-lukS*

PCR was performed to determine the presence of the *PVL* toxin gene *lukF-lukS* as previously described by Lina et al [36].

## Results

### 1. The overall antimicrobial susceptibility profiles of the bovine *S. aureus* isolates

The antibiotic susceptibility of the analyzed *S. aureus* isolates were shown in Table 1. The overall resistance rates were high with penicillin (97.1%) and sulfafurazole (83.5%), while the overall resistance rates were generally low with gentamicin (11.7%), ciprofloxacin (2.9%), cefazolin (6.8%), vancomycin (0%), and oxacillin (12.6%). The resistance rates were at moderate levels to tetracycline (35%), erythromycin (31.1%), and clindamycin (29.1%). Although there were variations in the resistance rates among the 4 regions, it was not obvious that one region showed more incidence of resistance than others (Table 1).

**Table 1:** The overall drug susceptibility patterns of the *S. aureus* isolates from bovine mastitis cases*.

		Penicillin		Gentamicin		Tetracycline		Erythromycin		Clindamycin		Ciprofloxacin		Sulfafurazole		Cefazolin		Vancomycin		Oxacillin	
Region#		R	S	R	S	R	S	R	S	R	S	R	S	R	S	R	S	R	S	R	S
Shanghai(52)	Number of isolates	52	0	11	37	23	29	17	35	24	21	3	48	49	3	6	46	0	52	11	41
	Percentage (%)	100	0	21.2	71.2	44.2	55.8	32.3	67.3	46.9	39.5	5.7	92.3	94.2	5.8	11.5	88.5	0	100	21.2	78.8
Sichuan(16)	Number of isolates	16	0	0	15	6	10	3	12	2	14	0	15	12	4	1	15	0	16	2	14
	Percentage (%)	100	0	0	93.75	37.5	62.5	18.75	75	14.3	85.7	0	93.75	75	25	6.67	93.33	0	100	12.5	87.5
Guizhou(18)	Number of isolates	18	0	0	18	4	14	7	11	2	16	0	18	12	6	0	18	0	18	0	18
	Percentage (%)	100	0	0	100	22.22	77.78	38.89	61.11	12.5	87.5	0	100	66.67	33.33	0	100	0	100	0	100
Gansu(17)	Number of isolates	14	1	1	16	3	13	5	6	2	10	0	17	13	3	0	17	0	17	0	17
	Percentage (%)	82.4	5.88	5.88	94.1	17.7	76.5	29.4	35.3	11.8	58.8	0	100	76.5	17.7	0	100	0	100	0	100
Total(103)	Number of isolates	100	1	12	86	36	66	32	64	30	61	3	98	86	16	7	96	0	103	13	90
	percentage (%)	97.1	0.97	11.7	83.5	34.95	64.1	31.1	62.1	29.1	59.2	2.9	95.1	83.5	15.5	6.8	93.2	0	100	12.6	87.4

* R: resistant; S: susceptible.

# The number in parenthesis indicate the total number of isolates from a given region.

### 2. Identification of *mec*A by PCR

**Figure 1:**
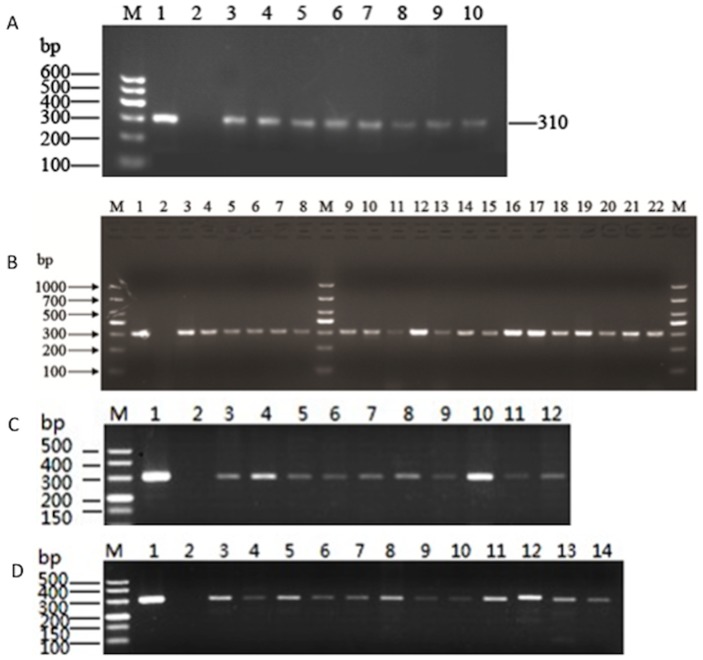
PCR detection of *mecA*-positive *S. aureus* isolates. The results were analyzed by Agarose gel electrophoresis. (A) *mecA*-positive isolates from Gansu province. Lane M: DNA size Marker. Lane 1: positive control strain ATCC43300. Lane 2: negative control strain ATCC25923. Lanes 3–10: isolate QY4, QY6, QY8, QY10, HG2, HG3, HG4 and HG5, respectively. (B) *mecA*-positive isolates from Shanghai. M: DNA size Marker. Lane 1: positive control strain ATCC43300. Lane 2: negative control strain ATCC25923. Lane 3–8: isolate SX5, SX6, SX10, SX11, SX13 and SX15, respectively; and lanes 9–22: isolates SH1, SH2, SH3, SH4, SH7, SH8, SH9, SH10, SH13, SH14, SH16, SH17, SH18 and SH20, respectively. (C) *mecA*-positive isolates from Guizhou. M: DNA size Marker. Lane 1positive control strain ATCC43300. Lane 2: negative control strain ATCC25923. Lanes 3–12: isolates zy1, zy2, zy4, zy5, zy6, zy8, zy11, zy12, zy14, and zy15, respectively. (D). *mecA*-positive isolates from Sichuan. M: DNA size Marker. Lane 1positive control strain ATCC43300. Lane 2: negative control strain ATCC25923. Lanes 3–14: isolates cx1, cx2, cx5, cx6, cx8, cx9, cx10, cx13, cx14, cx17, cx18, and cx19, respectively.

Using primers P1 and P2, we analyzed the presence of *mec*A in the *S. aureus* isolates. As shown in Fig. 1, the positive control strain ATCC43300 showed a distinct 310-bp band, while the negative control strain ATCC 25923 did not show a PCR product, indicating the specificity of the PCR assay. Among the *S. aureus* isolates examined in this study, 8, 20, 11, and 10 *mecA*-positive strains were identified for Gansu, Shanghai, Sichuan, and Guizhou, respectively (Fig. 1). The overall detection rate of *mecA* is 47.6% (49 out of 103), indicating the high prevalence of MRSA in *S. aureus* isolates derived from bovine mastitis in China.

### 3. Identification of OS-MRSA

According to the oxacillin disk diffusion tests, only 12.6% of the isolates were resistant to this antibiotic (Table 1), but 47.6% of the isolates were *mecA*-positive, suggesting the presence of OS-MRSA. In detail, all 17 *S. aureus* isolates from Gansu were susceptible to the antibiotic, but 8 (47.06%) of them were found carrying the *mecA* gene by PCR (Fig. 1; Table 2) and were classified as OS-MRSA. Four of the eight *mecA*-positive isolates were from farm A, and the other 4 from farm B. Among the 52 isolates from Shanghai, 11 were resistant to oxacillin. However, the PCR assay revealed that 20 of the 52 isolates were positive for *mecA* (Fig. 1). These *mecA*-positive isolates included 11 oxacillin-resistant strains and 9 oxacillin-susceptible strains (Table 3). Thus the 9 *mecA*-positive but oxacillin-susceptible isolates were classified as OS-MRSA. Among the 16 *S. aureus* isolates from Sichuan, 2 were resistant to oxacillin, but 11 were positive for *mecA* by PCR (Table 4), which included 1 oxacillin-resistant strain and 10 oxacillin-susceptible isolates (classified as OS-MRSA). Thus OS-MRSA accounted for 62.5% of the isolates from Sichuan. For the 18 isolates from Guizhou, none was resistant oxacillin, but 10 were positive for *mecA* and were considered OS-MRSA (Table 5). Among the 49 *mecA*-positive isolates, 37 were susceptible to oxacillin, indicating that the majority (75.5%) of the bovine MRSA are OS-MRSA. In total, OS-MRSA accounted for 35.9% (37 out of 103) of the total *S. aureus* isolates, indicating the high prevalence of OS-MRSA in clinical bovine mastitis cases in China.

**Table 2:** Genotyping and antibiotic susceptibility patterns of the *mecA*-positive *S. aureus* isolates from Gansu Province.

		OXA MIC			*ccr* gene		Resistance profile^b^								
Isolate^a^	*mecA*	(µg/mL)	*PVL*	*spa*	complex	SCC*mec*	PEN	GENTA	TETR	ERYTH	CLIN	CIP	SULF	CEFA	VAN
QY4	+	<0.5	−	t267	5	V	R	S	R	R	I	S	R	S	S
QY6	+	<0.5	−	t267	5	V	R	S	S	R	S	S	S	S	S
QY8	+	1	−	t267	5	V	R	S	S	I	R	S	R	S	S
QY10	+	0.5	−	t267	5	V	R	S	S	I	I	S	I	S	S
HG2	+	<0.5	−	t267	5	V	S	S	S	I	S	S	R	S	S
HG3	+	<0.5	−	t267	5	V	R	S	S	I	S	S	R	S	S
HG4	+	<0.5	−	t267	5	V	R	S	S	S	S	S	R	S	S
HG5	+	<0.5	−	t267	5	V	R	S	S	S	S	S	R	S	S

a QY and HG represent isolates from two different farms.

b R, resistant; S, susceptible.

OXA, Oxacillin ; PEN, penicillin; GENTA, gentamicin; TETR, tetracycline; ERYTH, erythromycin; CLIN, clindamycin ; CIP, ciprofloxacin; SULF, sulfamethoxazole; CEFA, cefazolin; VAN, vancomycin.

**Table 3:** Genotyping and antibiotic susceptibility profiles of the *mecA*-positive isolates identified in Shanghai.

		OXA MIC				Resistance profile^b^								
Isolates^a^	*mecA*	(µg/mL)	*PVL*	*spa*	SCC*mec*	PEN	GENTA	TETR	ERYTH	CLIN	CIP	SULF	CEFA	VAN
SX5	+	≥8	−	t1234	II	R	R	R	R	R	R	R	R	S
SX6	+	≥8	−	t1234	II	R	R	R	R	R	S	R	R	S
SX10	+	≥8	−	NT	V	R	R	R	S	R	R	R	R	S
SX11	+	≥8	−	NT	II	R	R	S	R	R	R	R	R	S
SX13	+	≥8	−	t267	V	R	R	R	R	R	S	R	R	S
SX15	+	≥8	−	t267	V	R	R	R	R	R	S	R	R	S
**SH1**	+	**≤1**	−	t267	V	R	S	S	S	R	S	R	S	S
SH2	+	≥8	−	t267	V	R	S	R	R	R	S	R	S	S
SH3	+	≥8	−	t267	V	R	S	R	R	R	S	R	S	S
**SH4**	+	**≤1**	−	t267	V	R	S	S	S	I	S	R	S	S
**SH7**	+	**≤1**	−	t267	V	R	S	S	S	I	S	R	S	S
SH8	+	≥8	−	t267	V	R	S	S	R	R	S	R	S	S
**SH9**	+	**≤1**	−	t267	V	R	S	S	S	R	S	R	S	S
**SH10**	+	**≤1**	−	t267	V	R	S	S	S	R	S	R	S	S
**SH13**	+	**≤1**	−	t1234	II	R	S	S	S	R	S	R	S	S
**SH14**	+	**≤1**	−	t1234	II	R	S	R	S	R	S	R	S	S
SH16	+	≥8	−	t1234	II	R	I	S	R	R	S	R	S	S
SH17	+	≥8	−	t1234	II	R	I	S	R	R	S	R	S	S
**SH18**	+	**≤1**	−	t1234	II	R	S	S	S	R	S	R	S	S
**SH20**	+	**≤1**	−	t1234	II	R	S	S	S	R	S	R	S	S

a SX and SH represent isolates from two different farms. Bold indicates OS-MRSA.

b R, resistant; S, susceptible.

OXA, Oxacillin ; PEN, penicillin; GENTA, gentamicin; TETR, tetracycline; ERYTH, erythromycin; CLIN, clindamycin ; CIP, ciprofloxacin; SULF, sulfamethoxazole; CEFA, cefazolin; VAN, vancomycin.

**Table 4:** Antibiotic susceptibility profiles of the *mecA*-positive isolates identified in Sichuan.

		OXA MIC	Resistance profile^a^								
Isolates	*mecA*	(µg/mL)	PEN	GENTA	TETR	ERYTH	CLIN	CIP	SULF	CEFA	VAN
CX1	+	<0.5	R	S	S	S	I	S	R	S	S
CX2	+	<0.5	R	S	S	S	S	S	S	S	S
CX5	+	≤1	R	S	R	R	R	S	R	S	S
CX6	+	≤2	R	S	S	S	I	S	R	S	S
CX8	+	<0.5	S	S	R	S	S	S	R	S	S
CX9	+	≥8	R	I	S	I	S	S	R	R	S
CX10	+	≤2	R	S	S	S	S	S	R	S	S
CX13	+	≤2	R	S	R	S	S	S	S	S	S
CX14	+	≤2	R	S	R	S	S	S	S	S	S
CX17	+	≤2	R	S	R	S	S	S	R	S	S
CX19	+	≤2	R	S	S	R	R	S	S	S	S

a R, resistant; S, susceptible.

OXA, Oxacillin ; PEN, penicillin; GENTA, gentamicin; TETR, tetracycline; ERYTH, erythromycin; CLIN, clindamycin ; CIP, ciprofloxacin; SULF, sulfamethoxazole; CEFA, cefazolin; VAN, vancomycin.

**Table 5:** Antibiotic susceptibility profiles of the *mecA*-positive isolates identified in Guizhou.

		OXA MIC	Resistance profile^a^								
Isolates	*mecA*	(µg/mL)	PEN	GENTA	TETR	ERYTH	CLIN	CIP	SULF	CEFA	VAN
ZY1	+	<0.5	R	S	R	S	I	S	R	S	S
ZY2	+	<0.5	R	S	S	S	S	S	R	S	S
ZY4	+	<0.5	R	S	S	R	R	S	S	S	S
ZY5	+	<0.5	R	S	S	S	I	S	R	S	S
ZY6	+	≤2	S	S	R	S	S	S	R	S	S
ZY8	+	<0.5	R	R	S	S	S	S	R	S	S
ZY11	+	≤2	R	S	S	R	S	S	S	S	S
ZY12	+	<0.5	R	S	R	S	S	S	R	S	S
ZY14	+	<0.5	R	S	S	R	S	S	S	S	S
ZY15	+	<0.5	R	S	S	S	S	S	R	S	S

a R, resistant; S, susceptible.

OXA, Oxacillin ; PEN, penicillin; GENTA, gentamicin; TETR, tetracycline; ERYTH, erythromycin; CLIN, clindamycin ; CIP, ciprofloxacin; SULF, sulfamethoxazole; CEFA, cefazolin; VAN, vancomycin.

### 4. Antimicrobial susceptibility patterns of the *mec*A-positive *S. aureus* isolates

Antibiotic susceptibility patterns of the *mecA*-positive isolates from different provinces are shown in Tables 2–5. For the *mecA*-positive isolates from Gansu, their oxacillin MICs were all lower than 2 µg/ml, consistent with the result from the disk diffusion test. They were also all susceptible to gentamicin, ciprofloxacin, cefazolin, and vancomycin, and most of them were non-resistant to tetracycline, erythromycin, and clindamycin (Table 2). However, the isolates were generally resistant to penicillin and sulfafurazole.

For the *mecA*-positive isolates from Shanghai (Table 3), 11 were resistant and 9 were susceptible to oxacillin, consistent with the result from the disk diffusion test. All of the 20 isolates were resistant to penicillin and sulfafurazole, but were susceptible to vancomycin. Most of them were also resistant to clindamycin. However, there were major differences in the resistance to other antibiotics between the isolates from farm SX and those from farm SH (Table 3). For example, all of the SX isolates were resistant to gentamycin and cefazolin, while all of the SH isolates were susceptible to the two antibiotics. Additionally, the SX isolate were more resistant to tetracycline, erythromycin, and ciprofloxacin than the SH isolates. These results suggest farm-to-farm variations in the antimicrobial susceptibility patterns.

Among the 11 *mecA*-positive isolates from Sichuan, 10 were OS-MRSA (oxacillin MICs<2 µg/ml) (Table 4). High resistance rates were observed with penicillin and sulfafurazole, but the isolates were generally susceptible to other tested antibiotics. The 10 *mecA*-carrying S. *aureus* from Guizhou were all susceptible to oxacillin (Table 5) and are considered OS-MRSA. Similar to the isolates from Sichuan, the Guizhou isolates were generally resistant to penicillin and sulfafurazole, but susceptible to other examined antibiotics (Table 5).

### 5. Molecular characterization of selected MRSA isolates

Molecular typing analysis was done with the *mecA*-positive isolates from Gansu and Shanghai. The ones from Gansu were *spa*-type t267 (allelic profile: 07-23-12-21-17-34-34-34-33-34), SCC*mec*-type V, *ccr* complex 5, and *PVL* negative (Table 2). The sequence of the SCC*mec* amplicon of isolate HG5 was determined and it was found that it shared 99% identity to SCC*mec*-type V sequences (GenBank Accession No. AB505629.1), further confirming the PCR result. For the MRSA from Shanghai, all were *PVL* negative; *spa* types included t1234 (8 isolates), t267(10 isolates), and two non-typeable; and their SCC*mec* types were II and V (Table 3). The isolates from Sichuan and Guizhou were not typed. These results suggest the genetic diversity of the *mecA*-positive isolates from cases of bovine mastitis.

## Discussion

In this study, we characterized *S. aureus* isolates from bovine mastitis milk samples collected from 4 different province/regions in China and identified the high prevalence of OS-MRSA. To our best knowledge, this is the first comprehensive investigation of OS-MRSA of bovine origin, following our initial report [37] on the presence of OS-MRSA on dairy farms in the Inner Mongolia region of China. Although *S. aureus* is a major cause of bovine mastitis, previously published reports revealed low prevalence of bovine MRSA, implying that MRSA was not commonly associated with mastitis [38]. However, most of previous studies were based on phenotypic tests for identifying MRSA, which may misidentify OS-MRSA as MSSA and underestimate the true prevalence of MRSA. In this study, 47.6% (49 out of 103) of the *S. aureus* isolates were found carrying *mecA*, which is unexpectedly higher than the highest reported incidence (17.5%) of MRSA from mastitic milk samples [39]. Presence of *mecA* is generally recognized as the most reliable method for detection of methicillin resistance, and *mecA*-positive *staphylococcal* strains are considered to be resistant to all β-lactam antibiotics [32]. Nevertheless, *S. aureus* that carry the *mecA* gene but appear phenotypically susceptible to oxacillin and vice versa have been reported recently [40]–[42] [37]. Thus, combination of genotypic and phenotypic tests is necessary to avoid false positive or false negative results in identifying MRSA.

All of the OS-MRSA isolates had an oxacillin MIC<2 µg/ml (Tables 2–5), indicating that presence of the *mecA* gene did not confer a high-level resistance to oxacillin. The reason for this phenotype remains to be elucidated. A recent study suggested that amino acid mutations in the FemXAB proteins (involved in cell wall synthesis) might contribute to the OS-MRSA phenotype [43], but the association of the mutations with the phenotype has not been formerly proven. Without testing the *mecA* gene, these isolates could be misclassified as MSSA based on the result of conventional antimicrobial susceptibility tests. OS-MRSA that carry *mecA* may result in the emergence of highly resistant MRSA under treatment with β-lactam antibiotics, which underscores the need for precautions when treating OS-MRSA infections [10] [42]. Due to this risk, treatment of OS-MRSA should avoid β-lactam antibiotics. As shown in Tables 2–5, the identified MRSAs were generally susceptible to other classes of antibiotics, such as gentamicin, ciprofloxacin, kanamycin, and vancomycin. Therefore, treatment of mastitis caused by OS-MRSA using non-β-lactam antibiotics may prevent the unwanted consequence of antibiotic resistance development.

Based on the results of molecular characterization, all the OS-MRSA isolates from Gansu belonged to *spa*-type t267, SCC*mec*-type V, and *ccrC* complex 5, and were *PVL* negative (Table 2), suggesting that these isolates may be a single lineage that was common in the surveyed region. However, the MRSA isolates from Shanghai belonged to different *spa* and SCC*mec* types (Table 3), suggesting their genetic diversity. Among the previously reported MRSA isolates associated with bovine mastitis, lineages with *spa*-type t267 and SCC*mec*-type V have not been identified [24]. However, a clinical MRSA isolate with the group V SCC*mec* element and t267 polymorphic X-region of *spa* was recovered in American University of Beirut Medical Center (AUB-MC) by Tokajian et al [44]. Another *mecA*-positive oxacillin-resistant *S. aureus* carrying SCC*mec* type V and *spa* type t267 was identified from an inpatient in England [45]. These reports plus findings from this study suggest that this lineage of MRSA may cause infections in both human and bovine and can be either susceptible or resistant to oxacillin. On the other hand, OS-MRSAs belonging to SCC*mec* types I, II, III IIIv, and IV were reported previously [10] [11], suggesting that MRSA of different genetic backgrounds could be phenotypically susceptible to oxacillin.

None of the OS-MRSA isolates identified in this study harbored the *PVL* gene. This finding is similar to that reported by Türkyilmaz et al. and Aires-de-Sousa et al. [26] [46]. In contrast, over 50% of bovine *S. aureus* isolates were determined to carry the *PVL* gene in another study [47]. *PVL* is a known virulence factor in *S. aureus* and is involved in the development of soft tissue infections [48]. The absence of *PVL* in the identified OS-MRSA isolates and their susceptibility to certain antimicrobial agents suggest that they may be eliminated from infected animals by appropriate antibiotic treatments.

## Conclusions

This study demonstrates a high incidence of OS-MRSA on dairy farms in different regions of China. These OS-MRSA isolates carried the *mecA* gene, were susceptible to oxacillin, and could be mistakenly identified as MSSA if testing of the *mecA* gene were not conducted. These results suggest that MRSA is more commonly associated with bovine mastitis than previously realized, which is the case at least in China. Findings from this study and the increasing reports of OS-MRSA in clinical settings underscore the need for genetic tests as well as phenotypic assays to accurately identify MRSA.
